# Bibliographical Notices

**Published:** 1847-01

**Authors:** 


### BIBLIOGRAPHICAL NOTICES.

*An Anatomical Description of the Diseases of the Organs of Circulation and Respiration.* By CHARLES EWOLD HASSE, M. D., *Prof. of Pathology and Clinical Medicine at the University of Zurich, etc. etc. Translated and Edited by* W. E. SWAINE, M. D., *Physician Extraordinary to H. R. H. the Duchess of Kent.* Philadelphia, Lea & Blanchard, 1846; 8vo., pp. 377.

This is one of the works published under the auspices of the "Sydenham Society"—a British association organized for the purpose of publishing rare works, which might not otherwise be given to the public, thereby promoting sound medical literature. The work by Dr. Hasse is the first of a series by that author, which, completed, will embrace the whole province of pathological anatomy. In so far as it already extends it is complete, the present volume being limited to the organs of circulation and respiration. The author does not confine himself exclusively to pathological anatomy, but considers in connection with the morbid appearances, the history of their development, and the processes of disease with which they are associated as causes and effects. The materials are derived from the author's extensive investigations, together with the latest and best established facts, ascertained and confirmed by others. It is a sterling work, and will be especially acceptable to those who are engaged in pathological inquiries. We purpose, in our next number, to publish an extract on a novel and interesting subject of pathology, which may serve as a specimen of the style and character of the volume.

---

*The United States Dissector, by* Wm. E. Horner, M. D., *Prof. of Anatomy in the University of Pennsylvania. Fourth edition, with numerous Illustrations. Edited by* Henry E. Smith, M. D. 8vo., pp. 431. Philadelphia, Lea & Blanchard, 1846.

Why a "United States Dissector" is not as good as a "Dublin" or a "London" Dissector, we confess we have never been able to see. Yet we would be sworn that ten students buy the "Dublin" or "London," where one will buy the "United States." Perhaps the young men fancy that an Irishman from "College Park," or a genuine Cockney subject furnish the only reliable Royal patterns, and that American anatomists do not altogether keep up with the fashion and improvements of the mother country.

This is a better book by one half than either of the above importations, and withal illustrated by numerous and handsome cuts, so clearly and accurately drawn, that every part of the anatomy is made intelligible; but we have very little faith in the publishers being able to get rid of the edition, for "who reads an American book?" F. H. H.

---

*Outlines of the Arteries; with short descriptions, designed for the use of Medical Students. Outlines of the Nerves, do. do.* By John Neil, A. M., M. D. *Prosector, &c. in the University of Pennsylvania.*

Two beautiful little volumes with the above titles, have recently been published by Barrington & Haswell, of Philadelphia. Medical students, for whom they were especially designed, will find them of material service in obtaining a clear apprehension of the distributions and anatomical relations of the arterial and nervous systems. Practitioners, will, also, find them useful for easy reference to refresh the memory, or to ascertain facts relating to particular cases as they occur in practice.

---

*Adulterations of various substances used in Medicine and the Arts, with the means of detecting them; intended as a manual for the Physician, the Apothecary and the Artisan.* By Lewis C. Beck, M. D., *Prof. of Chemistry in Rutger's College, New-Jersey, and in the Albany Medical College, &c. &c.* New-York, Samuel S. & William Wood, No. 261 Pearl-st., 1846. 12mo. pp. 333.

Dr. Beck deserves the thanks of the profession for the publication of this work. It has long been a desideratum. It should be owned by every apothecary, and will prove highly useful for reference to the practicing physician.

---

*American Medical Biography, embracing principally those eminent Physicians who have died since the publication of Dr. Thacher's work on the same subject.* By Stephen W. Williams, M. D., *late Prof. in the Berkshire, Willoughby, Fairfield, Ohio, and Hanover Med. Colleges, &c. &c.* 664 p. 8vo., with engravings; L. Merriam & Co. printers.

Surely he who chronicles the names and works of the virtuous dead does a goodly service, but we are especially bound to be thankful to him who seeks to rescue from oblivion the memory of those humble, but excellent members of our own profession, whose talents and lives have been expended in the zealous improvement of our science. For those who labor in the sevice of medicine there are few incentives to diligence, beyond the wants of nature and an approving conscience, since neither history nor marble have often recorded our names and acts, and in dying our remembrance has ceased forever. The man who invented an improved instrument of death, a shot missile, or a torpedo, is read in capitals, but he who discovered a machine to "repair the silver chord," and stay the ebbing tide of life, is forgotten in his country's record. In behalf of the profession, therefore, we give this work a hearty reception; it contains many examples which we shall hereafter strive well to emulate. The book is also illustrated with most excellent and life-like portraits of such men as Dewees, Hosack, Physick, Post, and others. F. H. H.

---

*Elements of Surgery.* By ROBERT LISTON, *Surgeon to the North London Hospital, &c.; Fourth American, from the last London Edition, with copious notes and additions.* By SAM'L D. GROSS, M. D., *Prof. of Surgery in the Medical Institute of Louisville, &c.; Illustrated with numerous engravings.* Philadelphia, Ed. Barrington & Geo. D. Haswell, 1846. 8 vo. pp. 660.

Liston is emphatically *the* surgical authority of the day. The number of his publications evince a laudable desire on his part, to contribute to the advancement and diffusion of surgical knowledge. The extensive additions by the editor, Prof. Gross, for the American student, greatly enhance the interest and value of the work. As a sound judicious surgeon, Prof. G. hold a high rank among the many eminent practitioners and teachers of this department of medical science and art.

---

*The Prescriber's Pharmacopeia; containing all the Medicines in the London Pharmacopeia, arranged in classes according to their action, with their composition and doses.* By a PRACTICING PHYSICIAN; *altered to correspond with the U. S. Dispensatory. Revised and improved by an American Physician. Second American from the third London Edition.* New-York, Samuel S. & Wm. Wood, No. 261 Pearl-st. 24 mo. pp. 144.

This is the title of a pocket volume, which will be found to contain much information of every-day utility to the medical practitioner.

